# Nirmatrelvir plus ritonavir remains effective in vaccinated patients at risk of progression with COVID-19: A systematic review and meta-analysis

**DOI:** 10.7189/jogh.13.06032

**Published:** 2023-07-21

**Authors:** Huamin Li, Huairong Xiang, Bei He, Qizhi Zhang, Wenxing Peng

**Affiliations:** 1 Department of Pharmacy, the Second Xiangya Hospital, Central South University, Changsha, Hunan, China; 2 Institute of Clinical Pharmacy, Central South University, Changsha, Hunan, China

## Abstract

**Background:**

The efficacy of nirmatrelvir plus ritonavir (NMV-r) for vaccinated COVID-19 patients at high risk of progression is not adequately recognised. To address this gap, we conducted a systematic review and meta-analysis of current literature.

**Methods:**

We searched PubMed, Web of Science, Embase, Cochrane Library, and medRxiv for articles published up to 8 January 2023 on NMV-r in outpatients. At least two researchers screened articles, extracted data, and assessed the quality of selected studies. We evaluated the results via risk ratios (RRs) with 95% confidence intervals (CIs) and tested for heterogeneity using *I*^2^ statistics.

**Results:**

We included seven observational cohort studies comprising 224 238 vaccinated patients. According to our meta-analysis, NMV-r reduced 47% incidence of all-cause death or hospitalisation within 30 days for vaccinated patients (RR = 0.53; 95% CI = 0.40-0.70; *I^2^* = 81%). After excluding the most influential result by sensitivity analysis, NMV-r still reduced risk of all-cause death or hospitalisation by 38% (RR = 0.62; 95% CI = 0.56-0.69; *I*^2^ = 0%). In our secondary outcome, NMV-r also showed its benefits in reducing all-cause death in vaccinated patients (RR = 0.40; 95% CI = 0.19-0.85; *I^2^* = 23%).

**Conclusions:**

We found positive evidence for the use of NMV-r for vaccinated patients at high-risk of progression with mild to moderate COVID-19. However, large-scale RCTs are needed to confirm these findings.

**Registration:**

PROSPERO CRD42023391349.

Novel agents for treatment of coronavirus disease 2019 (COVID-19) are becoming accessible as the pandemic progresses, including two FDA-approved oral therapeutics for mild to moderate patients: nirmatrelvir plus ritonavir (NMV-r) [1] and molnupiravir [2]. Both are favoured by clinicians, as they are administered orally, yet clinical trials showed NMV-r was more effective than molnupiravir in lowering hospitalisation or all-cause death compared to placebo (89% vs 30%) [3,4] without increasing the risk of teratogenicity or mutagenicity, making it more appropriate for patients with mild to moderate COVID-19 who are at high risk of progression.

NMV-r is an antiviral therapeutic developed by Pfizer for the treatment and post-exposure prophylaxis of COVID-19 [1]. Nirmatrelvir is the main protease inhibitor (Mpro) of severe acute respiratory syndrome coronavirus 2 (SARS-CoV-2) [5]; its level can be increased by the administration of ritonavir, a CYP3A inhibitor [6]. The United States encouraged the use of NMV-r to prevent hospital crowding in patients with risk factors for disease progression, regardless of vaccination status [7].

However, the evidence for the efficacy of NMV-r in vaccinated patients is insufficient. The EPIC-HR trial [4] (NCT04960202) demonstrated that NMV-r had an 89.1% risk reduction in COVID-19-related hospitalisation or death by 28 days from any cause in non-hospitalised, symptomatic adults (≥18 years). However, this phase 2-3, double-blind randomised trial only included unvaccinated patients with COVID-19. Only one real-world study [8] on vaccinated patients with COVID-19, found that NMV-r was associated with a reduced likelihood of emergency department visits, hospitalisation, or death. Two recent meta-analyses [9,10] found it had a beneficial effect in mild to moderate COVID-19 patients, but did not perform subgroup analyses.

Molnupiravir, however, did not reduce hospital admission or death in vaccinated adults at increased risk of adverse outcomes [11]. Considering general increase in vaccination rates, a better understanding of the clinical efficacy of NMV-r in vaccinated patients is required to guide individual and public health decisions. We aimed to provide a systematic review and meta-analysis of efficacy of NMV-r for vaccinated COVID-19 patients at high risk of progression.

## METHOD

### Registration

We registered the study protocol in the International Prospective Register of Systematic Reviews (PROSPERO) (registration number CRD42023391349) and reported our meta-analysis per the Preferred Reporting Items for Systematic Reviews and Meta-analysis (PRISMA) guidelines [12].

### Search strategy and selection criteria

Two researchers (HML, HRX) electronically searched PubMed, Embase, Web of Science, the Cochrane Library, and medRxiv for articles or preprints published between 1 January 2020 and 8 January 2023 (Table S1 in the **Online Supplementary Document**). Two researchers (HML, BH) separately screened the titles, abstracts, and full texts of selected studies using Revman (Cochrane Collaboration, version 5.4). We resolved disagreements by mutual consensus or consultations with a third researcher (HRX).

We included studies on non-hospitalised patients (≥12 years) with mild to moderate COVID-19 and at least one risk factor, studies stratifying patients into subgroup by vaccination, studies with data of one of outcomes (hospitalisation or all-cause death within 30 days and death in 30 days), studies on patients that used NMV-r within five days after testing positive, and studies where the control groups was treated without NMV-r, remdesivir, molnupiravir, and other monoclonal antibodies. We excluded case reports, letters, comments, and non-English articles.

### Data extraction and quality assessment

Two researchers (HML, HRX) individually extracted the following data from each eligible trial using a pre-designed form: author, year, type of study, publication status, country of enrolment, patient characteristics (age, sex, group, vaccinated group, and vaccination status), and outcomes of interest (only extracted outcome data stratified by vaccinated groups). The primary outcome was all-cause death or hospitalisation within 30 days and the secondary outcome was death within 30 days. We employed the Newcastle-Ottawa Scale (NOS) to evaluate the quality of included studies [13]. The scale includes nine items in three components (selection, comparability, and outcome), with each item scoring one point for a total of nine points. Two researchers (HML, HRX) independently scored the studies and the third researcher (BH) resolved discrepancies.

### Statistical analysis

We used the random effects model to estimate the pooled risk ratios (RRs) and corresponding 95% confidence intervals (95% CIs) for all outcomes of interest. We evaluated the statistical heterogeneity of the study outcomes using the *I*^2^ statistic, considering it substantial and critical when *I*^2^≥50% [14]. We qualitatively assessed for publication bias by visually inspecting funnel plots. We further performed sensitivity analyses to explore the causes of heterogeneity and subgroup analyses to determine if results were influenced by age, publication status, or vaccine status. We considered the results as statistically significant when *P* < 0.05. We conducted all statistical analyses in Revman 5.4.

## RESULTS

### Search results and characteristics of included articles

**Figure 1:**
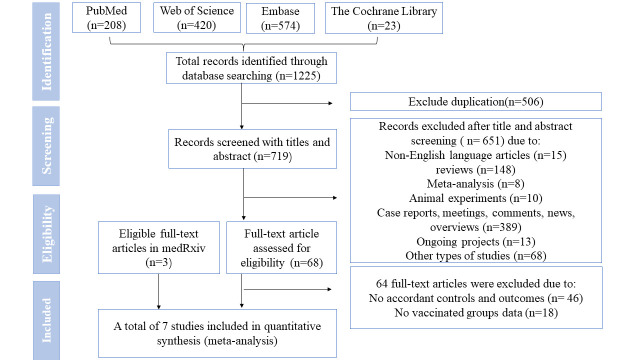
Study selection process. NMV-r – nirmatrelvir plus ritonavir.

The search retrieved 1225 articles, with 719 remaining after automated and manual deduplication. We excluded 651 articles through the title and abstract screening, including meta-analyses, reviews, non-English language studies, among others. Finally, we included seven retrospective cohort studies with 303 181 patients (**Figure 1**), of whom 224 238 were vaccinated and had at least one progression factors (chronic disease, disease of immune system, cancer, and so on). One study [15] included more than half of the overall sample of patients (66.99%). The two studies by Wong et. al. [16,17] contributed 8.91% and 0.09%, respectively. Zhou et. al. [18] and Dryden-Peterson et al. [19] accounted for 4.06% and 17.94%, while the studies by Ganatra et al. [8] and Bajema et al. [20] contributed 1.01% and 1.00% each (**Table 1**).

**Table 1:** Characteristics of studies included in the meta-analysis

Study, year	Type of study	Country	Publication status	NMV-r vs no NMV-r, n	Age in years – NMV-r vs no NMV-r	Females – NMV-r vs no NMV-r, n (%)	Vaccinated group – NMV-r vs no NMV-r, n (%)	Vaccination status	Contributions to a total of sample size, %
Bajema et al., 2022 [21]	OS	USA	Preprint	1587 vs 1587	65.0 (54.0-74.0), 66.0 (54.0-74.0)†	175 (11.0) vs 171 (10.7)	1126 (71.0) vs 1108 (69.8)	Not fully vaccinated	1.00
Dryden-Peterson et al., 2022 [19]	OS	USA	Publication	12 541 vs 32 010	NR§	7294 (58.0) vs 19 422 (61.0)	11 859 (91.0) vs 28 377 (91.0)	Fully vaccinated*	17.94
Ganatra et al., 2022 [8]	OS	USA	Publication	1130 vs 1130	57.5 (16.3), 57.7 (16.3)‡	712 (63.0) vs 724 (64.1)	1130 (100.0) vs 1130 (100.0)	Fully vaccinated	1.01
Wong et al., 2022 [16]	OS	China	Publication	890 vs 890	77.0 (14.2), 77.8 (15.8)‡	443 (49.8) vs 436 (49.0)	93 (10.4) vs 102 (11.5)	Fully vaccinated	0.09
Wong et al., 2022 [17]	OS	China	Publication	5542 vs 54 672	NR§	2976 (53.7) vs -29182 (53.4)	1850 (33.4) vs 18 138 (33.2)	Fully vaccinated	8.91
Zhou et al., 2022 [18]	OS	USA	Preprint	2808 vs 10 849	60.6 (15.8), 60.7 (15.8)‡	1625 (57.9) vs 6310 (58.2)	1897 (67.6) vs 7207 (66.4)	Not fully vaccinated	4.06
Schwartz et al. 2022 [15]	OS	Canada	Preprint	8876 vs 168 669	74.3, 74.4¶	5263 (59.3) vs 100 189 (59.4)	7527 (84.8) vs 142 694 (84.6)	Fully vaccinated	66.99

OS – observational study, USA – United States of America, NMV-r – nirmatrelvir plus ritonavir

*Fully vaccinated: received at least two doses of a vaccine.

†Age was expressed as medians and interquartile ranges.

‡Age was expressed as means and standard deviations.

§Age was presented in age groups only.

¶Age presented as means, but without standard deviations.

The studies usually had a roughly equal distribution of male and female patients, expect for the one by Bajema et al. [20]. The mean age of the patients was between 55 and 75 years, and most came from the United States of America and China. The vaccinated groups of Bajema et al. [20] and Zhou et al. [18] enrolled both patients who only received only one dose vaccine and fully vaccinated patients (received at least two doses vaccine), while the patients in the remaining five studies [8,15-17,19] were all fully immunised (**Table 1**). Four studies included information on hospitalisation or all-cause death within 30 days and data on deaths in 30 days (Table S2 in the **Online Supplementary Document**).

### Efficacy evaluation

#### Primary outcome

**Figure 2:**
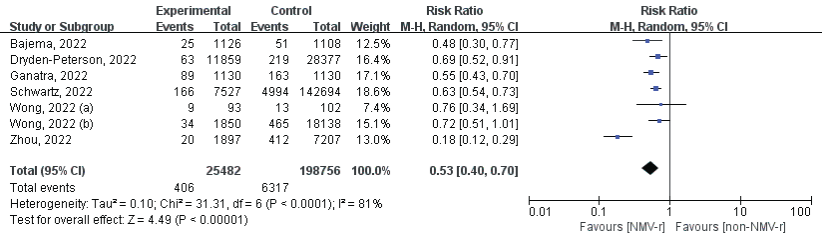
The forest plot for all-cause death or hospitalisation within 30 days. NMV-r – nirmatrelvir plus ritonavir.

All studies included data of primary outcome on 224 238 vaccinated patients, including 6723 events of all-cause death or hospitalisation during 30 fellow-up days. Results showed that using NMV-r within five days of the onset of symptoms could reduce 47% incidence of all-cause death or hospitalisation within 30 days for vaccinated patients (RR = 0.53; 95% CI = 0.40-0.70; *I*^2^ = 81%) (**Figure 2**). In the sensitivity analysis, we found significant bias in the data of Zhou et al. [18] with an RR of 18% (95% CI = 0.12-0.29), resulting in significant heterogeneity. We found no heterogeneity in the total sample size of 215 134 after excluding data from Zhou et al. [18], after which NMV-r decreased risk of all-cause death or hospitalisation by 38% (RR = 0.62, 95% CI = 0.56-0.69; *I*^2^ = 0%) (Figure S1 in the **Online Supplementary Document**).

**Figure 3:**
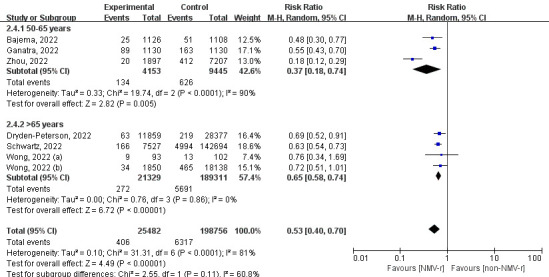
The forest plot of subgroup analysis of age for all-cause death or hospitalisation within 30 days. NMV-r – nirmatrelvir plus ritonavir.

**Figure 4:**
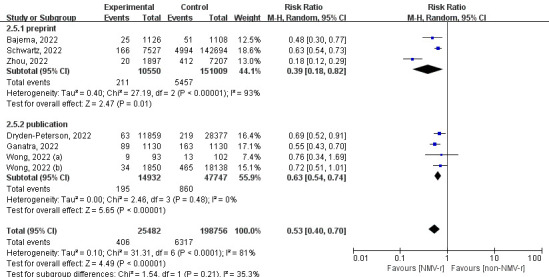
The forest plot of subgroup analysis of publication status for all-cause death or hospitalisation within 30 days. NMV-r – nirmatrelvir plus ritonavir.

**Figure 5:**
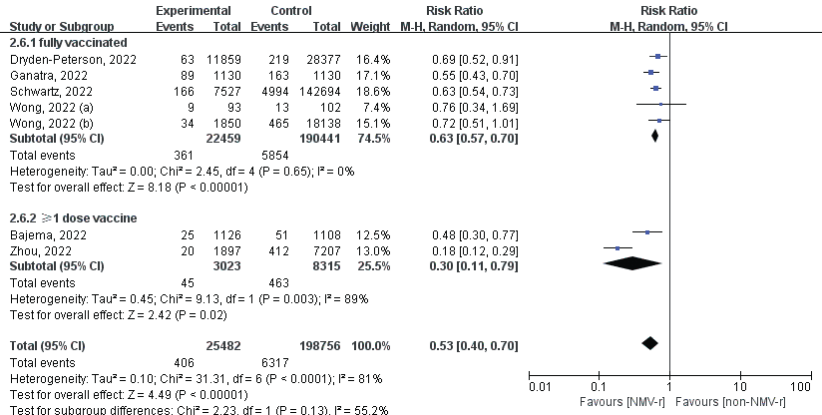
The forest plot of subgroup analysis of vaccinated status for all-cause death or hospitalisation within 30 days. NMV-r – nirmatrelvir plus ritonavir.

According to our subgroup analysis of different ages, NMV-r was more beneficial in preventing hospitalisation or death for COVID-19 patients aged 50-65 years (RR = 0.37) than for patients >65 years (RR = 0.65) (**Figure 3**). Additionally, the finding of the subgroup of publication status was consistent with the result of the sensitivity analysis, as NMV-r was associated with a 37% decrease in hospitalisation or all-cause death (RR = 0.63; 95% CI = 0.54-0.74) (**Figure 4**). Furthermore, NMV-r was more effective in patients who received one dose of vaccine or more (RR = 0.30) compared to fully vaccinated groups (RR = 0.63), but substantial heterogeneity existed (*I*^2^ = 89%) (**Figure 5**).

#### Secondary outcome

**Figure 6:**
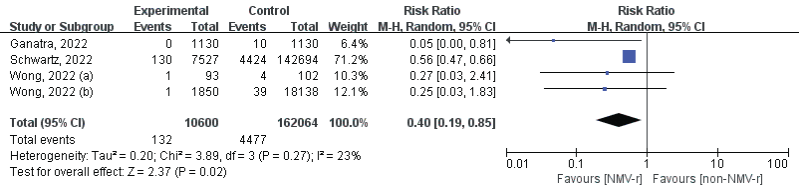
The forest plot for all-cause death within 30 days. NMV-r – nirmatrelvir plus ritonavir.

Four studies reported results of all-cause death during 30 days with a total of 172 664 fully vaccinated patients and an outcome of 4609 deaths. In fully vaccinated COVID-19 patients, NMV-r was able to reduce all-cause mortality by 60% (RR = 0.40; 95% CI = 0.19-0.85; *I*^2^ = 23%) without substantial heterogeneity (**Figure 6**).

### Quality of the evidence and risk of bias

According to the NOS analyses, exposure and non-exposure groups were representative in all studies, and the assessment of exposure factors and outcome indicators were recorded through electronic health records (Table S3 in the **Online Supplementary Document**). The accuracy of results of Wong et al. [17] and Schwartz et al. [15] was possibly affected because they failed to specify if the study controlled for the most important confounding factor. Most of the trials had a follow-up period of more than 30 days. The funnel plot suggested that our results could have been affected by publication bias or small sample studies. (Figure S2 in the **Online Supplementary Document**).

## DISCUSSION

To the best of our knowledge, this is the first meta-analysis on the use of NMV-r in vaccinated patients that used data from seven observational studies with a total of 224 238 vaccinated patients. According to our results, NMV-r lowered the risk of hospitalisation or death for vaccinated patients with mild to moderate COVID-19 compared to non-NMV-r groups. We found that NMV-r was more effective in patients aged 50-65 years than those >65 years. However, the effectiveness of NMV-r in vaccinated patients (RR = 47%) was lower than in unvaccinated patients in the EPIC-HR trial [4] (RR = 89.1%), which we believe was due to the protective effect of the vaccine. Even without population-wide coverage, COVID-19 vaccines have significantly reduced the number of disease related hospitalisations and deaths from SARS-CoV-2 in the USA [21]. However, our results still mean that the clinical benefits of NMV-r are greater than those provided by vaccination.

Sensitivity analysis highlighted that the study by Zhou et al. [18] had the greatest impact on the pooled volume and caused significant heterogeneity. There were statistically significant differences between the NMV-r and non-NMV-r group in the subgroup (≥85 years old) after using propensity score matching (PSM), meaning that the control group likely contained more people aged ≥85 years and that advanced age was also a factor of disease progression leading to hospitalisation or death.

Concerns regarding a rebound phenomenon have recently been expressed. According to case reports [22], three vaccinated patients above 60 years old tested positive at RT-PCR again after using NMV-r. But the study of Ganatra et al. [8] included in our meta-analysis showed no statistically significant difference for vaccinated patients between NMV-r group and control group. Furthermore, a recent study [23] found rebound occurred in 0.8% of patients, resulting in mild symptoms and no requirement for additional therapy. It is likely that some SARS-CoV-2 infections normally exhibit a viral load rebound and that the natural history of COVID-19 requires continued study [24].

NMV-r, as one of possible oral antiviral medicines, is probably more suitable for treating mild to moderate vaccinated patients, as molnupiravir did not reduce hospitalisation or death in vaccinated adults at increased risk of an adverse outcome [11]. However, most COVID-19 patients with risk factors for disease progression are treated with other drugs, and the possible effects of combination therapy must be considered cautiously [25].

### Strengths and limitations

Our results were limited by a lack of published data on the effectiveness of NMV-r for vaccinated patients. Additionally, 66.99% of the populations included in our analysis were patients from the study by Schwartz et al. [15], which could have biased our results. High-quality RCTs are required to confirm our findings.

Furthermore, there was no proof of NMV-r’s safety for COVID-19 patients. According to EPIC-HR [4], dysgeusia, diarrhoea, and vomiting were the most frequent adverse effects occurring in NMV-r recipients. The meta-analysis by Zheng et al. [9] found no significant difference of adverse events in NMV-r group and control group. Most of the included trials had a short follow-up period (within 30 days), so we were unable to determine if long-term sequelae or drug resistance issues existed to restrict the usage of NMV-r.

However, our study is the first meta-analysis of the efficacy of NMV-r in vaccinated patients. With the increase in NMV-r production and worldwide prevalence, our findings offer support for prescribing it to outpatients, as well as vaccinated individuals.

## CONCLUSIONS

Our findings support the use of NMV-r in the treatment of vaccinated patients with mild to moderate COVID-19 who are at high risk of progression. However, large-scale RCTs are needed to confirm these findings.

## Additional material


Online Supplementary Document
