# Supplementary material for: Nirmatrelvir plus ritonavir remains effective in vaccinated patients at risk of progression with COVID-19: A systematic review and meta-analysis
Source: J Glob Health. 2023 Jul 21;13:06032. doi: 10.7189/jogh.13.06032 (PMC10357131; doi:10.7189/jogh.13.06032)
Supplement: Online Supplementary Document [file jogh-13-06032-s001.pdf]

ONLINE SUPPLEMENTARY DOCUMENT

Title: Nirmatrelvir plus ritonavir remains effective in vaccinated patients at risk of progression with COVID-19: a systematic review and meta-analysis

Authors: Huamin Li, Huairong Xiang, Bei He, Qizhi Zhang, Wenxing Peng

Table S1: The search details

Table S2: The data on outcomes

Table S3: Quality assignments based on the Newcastle-Ottawa Scale

Figure S1: The forest plot of sensitive analysis for all-cause death or hospitalization within 30 days

Figure S2: The funnel plot of all-cause death or hospitalization within 30 days

Table S1: The search details

Table S1a: The search details of PubMed

#1	"nirmatrelvir and ritonavir drug combination"[Supplementary Concept] OR "nirmatrelvir and ritonavir drug combination"[All Fields] OR "nirmatrelvir and ritonavir drug combination"[Supplementary Concept] OR "nirmatrelvir and ritonavir drug combination"[All Fields] OR "pf 07321332 ritonavir"[All Fields] OR "nirmatrelvir and ritonavir drug combination"[Supplementary Concept] OR "nirmatrelvir and ritonavir drug combination"[All Fields] OR "nirmatrelvir ritonavir"[All Fields] OR "nirmatrelvir and ritonavir drug combination"[Supplementary Concept] OR "nirmatrelvir and ritonavir drug combination"[All Fields] OR "nirmatrelvir and ritonavir drug combination"[Supplementary Concept] OR "nirmatrelvir and ritonavir drug combination"[All Fields] OR "nirmatrelvir and ritonavir drug combination"[Supplementary Concept] OR "nirmatrelvir and ritonavir drug combination"[All Fields] OR "paxlovid"[All Fields] OR "nirmatrelvir and ritonavir drug combination"[Supplementary Concept]
#2	"COVID-19"[MeSH Terms] OR ("COVID-19"[All Fields] OR "COVID-19"[MeSH Terms] OR "covid 19 vaccines"[All Fields] OR "covid 19 vaccines"[MeSH Terms] OR "covid 19 serotherapy"[All Fields] OR "covid 19 nucleic acid testing"[All Fields] OR "covid 19 nucleic acid testing"[MeSH Terms] OR "covid 19 serological testing"[All Fields] OR "covid 19 serological testing"[MeSH Terms] OR "covid 19 testing"[All Fields] OR "covid 19 testing"[MeSH Terms] OR "sars cov 2"[All Fields] OR "sars cov 2"[MeSH Terms] OR "severe acute respiratory syndrome coronavirus 2"[All Fields] OR "ncov"[All Fields] OR "2019 ncov"[All Fields] OR (("coronavirus"[MeSH Terms] OR "coronavirus"[All Fields] OR "cov"[All Fields]) AND 2019/11/01:3000/12/31[Date - Publication]) OR ("COVID-19"[MeSH Terms] OR "COVID-19"[All Fields] OR "2019 ncov infection"[All Fields]) OR ("COVID-19"[MeSH Terms] OR "COVID-19"[All Fields] OR "2019 ncov infection"[All Fields]) OR ("COVID-19"[MeSH Terms] OR "COVID-19"[All Fields] OR "2019 ncov infections"[All Fields]) OR ("COVID-19"[MeSH Terms] OR "COVID-19"[All Fields] OR "infection 2019 ncov"[All Fields]) OR ("COVID-19"[MeSH Terms] OR "COVID-19"[All Fields] OR "sars cov 2 infection"[All Fields]) OR ("COVID-19"[MeSH Terms] OR "COVID-19"[All Fields] OR "infection sars cov 2"[All Fields]) OR ("COVID-19"[MeSH Terms] OR "COVID-19"[All Fields] OR "sars cov 2 infection"[All Fields]) OR ("COVID-19"[MeSH Terms] OR "COVID-19"[All Fields] OR "sars cov 2 infections"[All Fields]) OR ("COVID-19"[MeSH Terms] OR "COVID-19"[All Fields] OR "2019 novel coronavirus disease"[All Fields]) OR ("COVID-19"[MeSH Terms] OR "COVID-19"[All Fields] OR "2019 novel coronavirus infection"[All Fields]) OR ("COVID-19"[MeSH Terms] OR "COVID-19"[All Fields]

	OR "covid 19 virus infection"[All Fields]) OR ("COVID-19"[MeSH Terms] OR "COVID-19"[All Fields] OR "covid 19 virus infection"[All Fields]) OR ("COVID-19"[MeSH Terms] OR "COVID-19"[All Fields] OR "covid 19 virus infections"[All Fields]) OR ("COVID-19"[MeSH Terms] OR "COVID-19"[All Fields]) OR ("COVID-19"[MeSH Terms] OR "COVID-19"[All Fields] OR "virus infection covid 19"[All Fields]) OR ("COVID-19"[MeSH Terms] OR "COVID-19"[All Fields] OR "covid19"[All Fields]) OR ("COVID-19"[MeSH Terms] OR "COVID-19"[All Fields] OR "coronavirus disease 2019"[All Fields]) OR ("COVID-19"[MeSH Terms] OR "COVID-19"[All Fields] OR "disease 2019 coronavirus"[All Fields]) OR ("COVID-19"[MeSH Terms] OR "COVID-19"[All Fields] OR "coronavirus disease 19"[All Fields]) OR ("COVID-19"[MeSH Terms] OR "COVID-19"[All Fields] OR "coronavirus disease 19"[All Fields]) OR ("COVID-19"[MeSH Terms] OR "COVID-19"[All Fields] OR "severe acute respiratory syndrome coronavirus 2 infection"[All Fields]) OR ("COVID-19"[MeSH Terms] OR "COVID-19"[All Fields] OR "covid 19 virus disease"[All Fields]) OR ("COVID-19"[MeSH Terms] OR "COVID-19"[All Fields] OR "covid 19 virus disease"[All Fields]) OR ("COVID-19"[MeSH Terms] OR "COVID-19"[All Fields]) OR ("COVID-19"[MeSH Terms] OR "COVID-19"[All Fields] OR "disease covid 19 virus"[All Fields]) OR ("COVID-19"[MeSH Terms] OR "COVID-19"[All Fields] OR "virus disease covid 19"[All Fields]) OR ("COVID-19"[MeSH Terms] OR "COVID-19"[All Fields] OR "sars coronavirus 2 infection"[All Fields]) OR ("COVID-19"[MeSH Terms] OR "COVID-19"[All Fields] OR "2019 ncov disease"[All Fields]) OR ("COVID-19"[MeSH Terms] OR "COVID-19"[All Fields] OR "2019 ncov disease"[All Fields]) OR ("COVID-19"[MeSH Terms] OR "COVID-19"[All Fields] OR "2019 ncov diseases"[All Fields]) OR ("COVID-19"[MeSH Terms] OR "COVID-19"[All Fields] OR "disease 2019 ncov"[All Fields]) OR ("COVID-19"[MeSH Terms] OR "COVID-19"[All Fields] OR "covid 19 pandemic"[All Fields]) OR ("COVID-19"[MeSH Terms] OR "COVID-19"[All Fields] OR "covid 19 pandemic"[All Fields]) OR ("COVID-19"[MeSH Terms] OR "COVID-19"[All Fields] OR "pandemic covid 19"[All Fields]) OR ("COVID-19"[MeSH Terms] OR "COVID-19"[All Fields] OR "covid 19 pandemics"[All Fields]))
#3	#1 AND #2 (limited time from 1,1,2020 to 1,8, 2023)
Total	208

Table S1b: The search details of Web of Science

#1	(((((TS=(Paxlovid)) OR TS=(nirmatrelvir, ritonavir drug combination)) OR TS=(PF-07321332, ritonavir)) OR TS=(nirmatrelvir, ritonavir)) OR TS=(PF-07321332 and ritonavir drug combination)) OR TS=(PF-07321332, ritonavir drug combination)
#2	(((((((((((((((((((((((((((((((((((TS=(COVID-19)) OR TS=(COVID 19)) OR TS=(2019-nCoV Infection))) OR TS=(2019 nCoV Infection)) OR TS=(2019-nCoV Infections)) OR TS=(Infection, 2019-nCoV)) OR TS=(SARS-CoV-2 Infection)) OR TS=(Infection, SARS-CoV-2)) OR TS=(SARS CoV 2 Infection)) OR TS=(SARS-CoV-2 Infections)) OR TS=(2019 Novel Coronavirus Disease)) OR TS=(2019 Novel Coronavirus Infection)) OR TS=(COVID-19 Virus Infection)) OR TS=(COVID 19 Virus Infection)) OR TS=(COVID-19 Virus Infections)) OR TS=(Infection, COVID-19 Virus)) OR TS=(Virus Infection, COVID-19)) OR TS=(COVID19)) OR TS=(Coronavirus Disease 2019)) OR TS=(Disease 2019, Coronavirus)) OR TS=(Coronavirus Disease-19)) OR TS=(Coronavirus Disease 19)) OR TS=(Severe Acute Respiratory Syndrome Coronavirus 2 Infection)) OR TS=(COVID-19 Virus Disease)) OR TS=(COVID 19 Virus Disease)) OR TS=(COVID-19 Virus Diseases)) OR TS=(Disease, COVID-19 Virus)) OR TS=(Virus Disease, COVID-19)) OR TS=(SARS Coronavirus 2 Infection)) OR TS=(2019-nCoV Disease)) OR TS=(2019 nCoV Disease)) OR TS=(2019-nCoV Diseases)) OR TS=(Disease, 2019-nCoV)) OR TS=(COVID-19 Pandemic)) OR TS=(COVID 19 Pandemic)) OR TS=(Pandemic, COVID-19)) OR TS=(COVID-19 Pandemics)
#3	#1 AND #2 (limited time from 1,1,2020 to 1,8, 2023)
Total	420

Table S1c: The search details of Embase

#1	covid AND 19 OR ('2019 ncov' AND infection) OR (2019 AND ncov AND infection) OR ('2019 ncov' AND infections) OR (infection, AND '2019 ncov') OR ('sars cov 2' AND infection) OR (infection, AND 'sars cov 2') OR (sars AND cov AND 2 AND infection) OR ('sars cov 2' AND infections) OR (2019 AND novel AND coronavirus AND disease) OR (2019 AND novel AND coronavirus AND infection) OR ('covid 19' AND virus AND infection) OR (covid AND 19 AND virus AND infection) OR ('covid 19' AND virus AND infections) OR (infection, AND 'covid 19' AND virus) OR (virus AND infection, AND 'covid 19') OR covid19 OR (coronavirus AND disease AND 2019) OR (disease AND 2019, AND coronavirus) OR (coronavirus AND 'disease 19') OR (coronavirus AND disease AND 19) OR (severe AND acute AND respiratory AND syndrome AND coronavirus AND 2 AND infection) OR ('covid 19' AND virus AND disease) OR (covid AND 19 AND virus AND disease) OR ('covid 19' AND virus AND diseases) OR (disease, AND 'covid 19' AND virus) OR (virus AND disease, AND 'covid 19') OR (sars AND coronavirus AND 2 AND infection) OR ('2019 ncov' AND disease) OR (2019 AND ncov AND disease) OR ('2019 ncov' AND diseases) OR (disease, AND '2019 ncov') OR ('covid 19' AND pandemic) OR (covid AND 19 AND pandemic) OR (pandemic, AND 'covid 19') OR ('covid 19' AND pandemics)
#2	paxlovid AND nirmatrelvir, AND ritonavir AND drug AND combination OR ('pf 07321332,' AND ritonavir) OR (nirmatrelvir, AND ritonavir) OR ('pf 07321332' AND ritonavir AND drug AND combination) OR ('pf 07321332,' AND ritonavir AND drug AND combination)
#3	#1 AND #2 (limited time from 1,1,2020 to 1,8, 2023)
Total	574

Table S1d: The search details of the Cochrane Library

#1	(Paxlovid) OR (nirmatrelvir, ritonavir drug combination) OR (PF-07321332, ritonavir) OR (nirmatrelvir, ritonavir) OR (PF-07321332 and ritonavir drug combination) OR (PF-07321332, ritonavir drug combination)
#2	(COVID-19) OR (SARS-CoV-2 Infection) OR (COVID-19 Virus Disease) OR (Coronavirus Disease 2019) OR (COVID-19 Pandemics)
#3	#1 AND #2 (limited time from 1,1,2020 to 1,8, 2023 and limited on trials)
Total	23

eTable 2: The data on outcomes

eTable 2a: The data on all-cause death or hospitalization within 30 days

Study	events	total	events	total
Bajema, 2022	25	1126	51	1108
Dryden-Peterson, 2022	63	11859	219	28377
Ganatra, 2022	89	1130	163	1130
Schwartz, 2022	166	7527	4994	142694
Wang, 2022 (a)	9	93	13	102
Wang, 2022 (b)	34	1850	465	18138
Zhou, 2022	20	1897	412	7207

eTable 2b: The data on all-cause death within 30 days

Study	events	total	events	total
Ganatra, 2022	0	1130	10	1130
Schwartz, 2022	130	7527	4424	142694
Wang, 2022 (a)	1	93	4	102
Wang, 2022 (b)	1	1850	39	18138

Table S3: Quality assignments based on the Newcastle-Ottawa Scale

study	selection				comparability			outcome	
	representativeness	selection of non-exposed	ascertainment of exposure	outcome not present at start	comparability on most important factors	comparability on other risk factors	assessment of outcome	long enough follow-up (median>30 days)	adequecy (completeness) of follow-up
Bajema, 2022	1	1	1	1	1	1	1	1	1
Dryden-Peterson, 2022	1	1	1	1	1	1	1	0	1
Ganatra,2022	1	1	1	1	1	1	1	1	1
Wong, 2022 (a)	1	1	1	1	1	0	1	1	1
Wong, 2022 (b)	1	1	1	1	0	1	1	1	1
Zhou, 2022	1	1	1	1	1	0	1	1	1
Schwartz, 2022	1	1	1	1	0	0	1	1	1

Study or Subgroup	paxlovid Events	paxlovid Total	Control Events	Control Total	Weight	Risk Ratio M-H, Fixed, 95% CI
Bajema, 2022	25	1126	51	1108	5.5%	0.48 [0.30, 0.77]
Dryden-Peterson, 2022	63	11859	219	28377	13.7%	0.69 [0.52, 0.91]
Ganatra, 2022	89	1130	163	1130	17.3%	0.55 [0.43, 0.70]
Schwartz, 2022	166	7527	4994	142694	53.1%	0.63 [0.54, 0.73]
Wang, 2022 (a)	9	93	13	102	1.3%	0.76 [0.34, 1.69]
Wang, 2022 (b)	34	1850	465	18138	9.1%	0.72 [0.51, 1.01]
**Total (95% CI)**		**23585**		**191549**	**100.0%**	**0.63 [0.56, 0.70]**
Total events	386		5905			

Heterogeneity: Chi² = 3.64, df = 5 (P = 0.60); I² = 0%
Test for overall effect: Z = 8.55 (P < 0.00001)

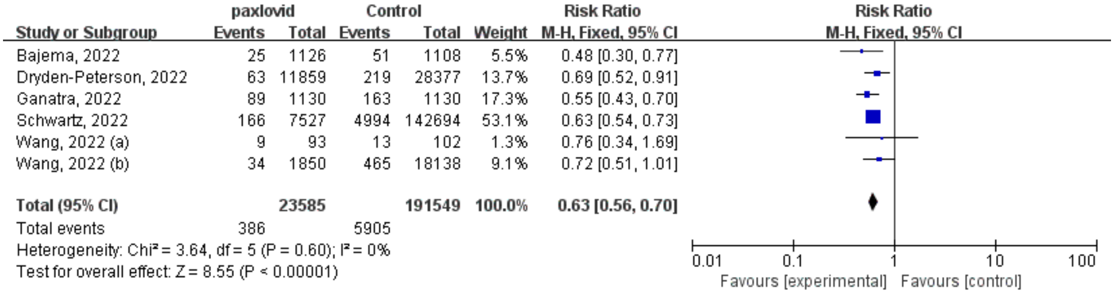

Figure S1: The forest plot of sensitive analysis for all-cause death or hospitalization within 30 days

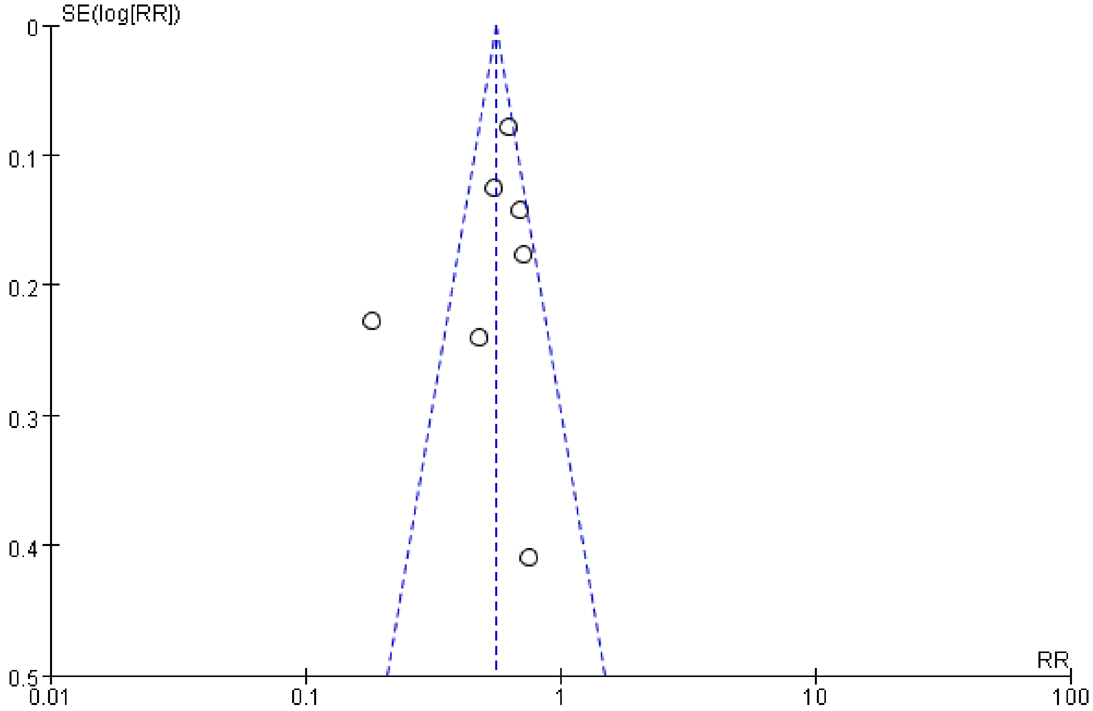

Figure S2: The funnel plot of all-cause death or hospitalization within 30 days
